# Change in waist circumference and lifestyle habit factors as a predictor of metabolic risk among middle-aged and elderly Japanese people: population-based retrospective 10-year follow-up study from 2008 to 2017

**DOI:** 10.1186/s13690-022-00836-z

**Published:** 2022-03-09

**Authors:** Haruko Ono, Kotomi Akahoshi, Michiaki Kai

**Affiliations:** 1 Department of Community Health Nursing, Oita University of Nursing and Health Sciences, 2944-9 Megusuno, 870-1201 Oita, Japan; 2 Oita University of Nursing and Health sciences, Oita, Japan

**Keywords:** Waist circumference, Lifestyle habits, Health checkup

## Abstract

**Background:**

Waist circumference (WC) increases more than body mass index (BMI) over time. This study investigated the change in WC among middle-aged and elderly Japanese people for 10 years, and its relationship with lifestyle and lipid metabolism factor.

**Methods:**

Health checkup data and lifestyle habits of a retrospective cohort of 745 people aged 40–65 years who underwent health checkups at least three times between 2008 and 2017 were analyzed. Information of Lifestyle habits about smoking history, regular exercise, alcohol intake skipping breakfast was collected using a self-administered questionnaire. Participants who were taking medications for diabetes, hyperlipidemia, or hypertension were excluded from analyses. Longitudinal associations between the change in WC and lifestyle habit factors with adjustments for sex, age, and WC at the start of health checkups were assessed using generalized linear models.

**Results:**

Regardless of lifestyle, body weight (BW) decreased 0.8 kg (*p* < 0.001) for women, 0.9 kg (*p* = 0.003) for men, WC increased 0.8 cm (*p* = 0.007) for women, 0.2 cm (*p* = 0.657) for men. In addition, serum triglycerides and high- and low-density lipoprotein levels estimated 10 years later revealed that increased WC ratios also exacerbated the respective blood sample data.

**Conclusion:**

Both men and women showed an increase in WC regardless of BW changes, and the increase in WC worsened lipid metabolism. For the middle-aged and elderly, whose WC increases over time, it will be more important to take notice of their WC than BW or BMI for effective health checkups.

## Background

In recent years, the increase in obesity has become a serious issue in Japan, and the increase in lifestyle-related diseases caused by obesity has caused problems such as rising medical costs. It is reported that Japan’s national medical expenses for the 2019 fiscal year was 40 trillion yen, of which more than 35% was used for lifestyle-related. To solve these issues, in 2008, the Japanese government started a new annual health checkup program called “Specialized Health Checkups” [1] to support early diagnosis and intervention in metabolic syndrome for all Japanese citizens aged 40–74 years. However, the prevalence of obesity among middle-aged men in Japan remains high at about 40% [2].

Body mass index (BMI) is used as a global standard as an indicator of obesity. One of the disadvantages of using BMI is that it does not distinguish body fat mass from muscle mass [3]. The composition of body fat mass and muscle mass varies by age, sex, and ethnicity [4, 5]. Women have a higher percentage of body fat than men with the same BMI [6]. Older adults have reduced muscle mass and, thus, increased fat mass [7]. Even in people with a BMI within the normal range, it has been suggested to be strongly associated with metabolic and cardiovascular disorders compared to total adiposity [8, 9].

It is getting more notice that waist circumference (WC) is a valuable measurement [10, 11]. Many studies have shown that WC is a better predictor of adverse health outcomes than is BMI [12]. Previous studies, mainly conducted in the USA, have shown that WC has increased above and beyond what would be anticipated based on secular trends in BMI [13–17]. In Japan, the prevalence of obesity is correlated with WC, especially as age increases [18]. A longitudinal study in the United Kingdom showed that secular increases in mid-adulthood WC are greater than BMI [19]. This suggests that health management based on WC is important for older adults in midlife and beyond.

Factors associated with abdominal obesity have been found to be related to lifestyle [20]. We hypothesized that long-term unhealthy lifestyle would increase WC more in middle age and beyond. In this study, the purpose of this study aimed to determine the association between changes in WC and lifestyle habits by tracking data from a population-based cohort of residents’ health examinations in the city of A in Japan from 2008 to 2017. The health examinations are conducted in communities and workplaces under the law. The medical checkup items are defined by law. The secondary objective of our study is to estimate the effect of the change to WC on metabolic risk.

## Materials and methods

### Participants

**Fig. 1:**
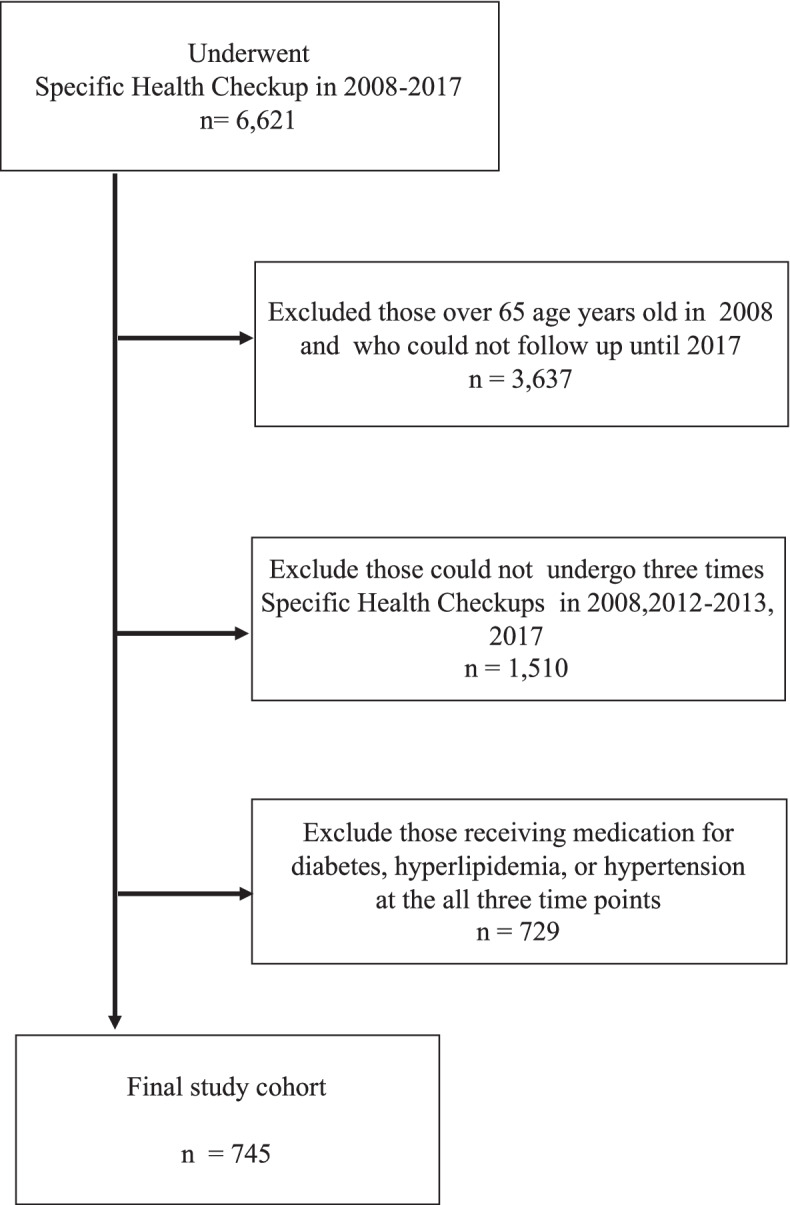
Flowchart for the inclusion and exclusion of this study cohort: Population-based retrospective study of Japan from 2008 to 2017 in Japan

A population-based longitudinal and individualized observational study was conducted using the Specialized Health Checkups data of 6,621 residents of a city in Oita Prefecture, Japan who were registered in the National Health Insurance in 2008. Data were collected and collated annually from 2008 to 2017. We extracted the data of 2,984 residents between the ages of 40 and 64 years. Of these, 1,474 residents had received specialized health checkups at least three times in 2008, 2012/13, and 2017. After excluding those who were receiving medication for diabetes, hyperlipidemia, or hypertension at the all three time points, 745 residents were included in this study (Fig. 1).

### Specialized health checkups in Japan

Specialized health checkups include annual laboratory tests, questionnaires, and a physical examination to evaluate metabolic syndrome risk factors. The laboratory tests and physical examination included measurements of WC, body weight (BW), BMI, systolic blood pressure (SBP), diastolic blood pressure (DBP), HbA1c, blood glucose, triglyceride (TG), high-density lipoprotein (HDL) cholesterol, and low-density lipoprotein (LDL) cholesterol [21]. The questionnaire assessed smoking status and lifestyle habits and whether participants took medication for diabetes, hypertension, or dyslipidemia. Measurement methods, diagnostic criteria, and protocols are described in the “Operational Guide to Specialized Health Checkups and the Specialized Health Guidance” by the Ministry of Health, Labor, and Welfare [21]. Participants in checkups, aged 40 to 74 years, were initially classified and assessed by obesity indicators (WC and BMI) and then by the number of additional metabolic risk factors.

### Data collection

The annual specialized health checkups were performed by well-trained examiners and included questions about medical history and anthropometric measurements. Height (without shoes) was measured to the nearest 0.1 cm using a standard stadiometer. Body weight was measured using a standard scale to the nearest 0.1 kg. BMI was calculated by dividing body weight (kg) by height squared (m^2^). WC was determined during minimal respiration in a standing position to the nearest 0.1 cm by measuring at the umbilical level using a flexible anthropometric tape. SBP and DBP were measured in a sitting position after participants had rested for at least 5 min. After a 12-h overnight fast, blood samples were drawn to measure TG, LDL, and HDL levels. A self-reported questionnaire based on a specialized health examination was used to collect information on lifestyle habits. The questions asked about their smoking history (≥ 100 cigarettes in the past year), regular exercise (at least 30 min, three times a week), near-daily alcohol intake, and skipping breakfast. Responses were categorized as “yes” or “no”.

### Statistical analysis

The means and standard deviations for all continuous variables and frequencies and percentages of categorical variables were calculated. Paired t-tests and McNemar tests were conducted to compare the 2008 and 2017 data. Correlations were assessed using Pearson’s correlation coefficients for WC, BMI, and body weight ratios.

Longitudinal associations between the change in WC and lifestyle habit factors with adjustments for sex, age, and WC at the start of specialized health checkups were assessed using generalized linear models (GLM). A GLM was fitted with the logarithm of the ratio of WC17 in 2017 to WC08 in 2008 as a response variable and a set of explanatory variables. Another GLM was fitted with the logarithm of the ratio of TG17 in 2017 to TG08 in 2008 as a response variable and a set of explanatory variables. The offset term, WC08 or TG08, was added to the models described above.

The size of WC at the start of specialized health checkups was based on the Japanese diagnostic criteria for metabolic syndrome: ≥ 85 cm for men and ≥ 90 cm for women [21]. Each lifestyle variable was dichotomized as follows: habits continued for more than five years = 1 and less than five years = 0. The predicted values of TG, HDL, and LDL were calculated from the fitted models obtained by the GLM analysis. The analysis used data from 729 participants, excluding 16 participants who had lifestyle-related missing data.

Data were analyzed using R software (R Foundation for Statistical Computing, Vienna, Austria) [22]. Statistical significance was set at *P* < 0.05.

### Ethics statement

This study was approved by the Research Ethics Safety Committee at Oita University of Nursing and Health Sciences before implementation (no. 18–69). The study fell under the category of “ethical guidance related to epidemiological surveys” since it used health data. The health checkup data received from B-City did not include any information that could identify the participating individuals. There was no negative impact on the participants by agreeing or declining to participate in the study, and there were no issues concerning the protection of human rights.

## Results

Participants’ baseline characteristics by age group and sex are presented in Table 1. The WC for men and women were 84.6 ± 8.5 cm and 79.1 ± 8.5 cm, respectively. The TG, HDL and LDL for men were 126.8 ± 70.4 mg/dl and 59.8 ± 16.4 mg/dl, 130.0 ± 30.2 mg/dl, respectively. The TG, HDL and LDL for women were 98.8 ± 48.5 mg/dl and 73.1 ± 18.3 mg/dl, 134.3 ± 29.2 mg/dl, respectively. Women had lower levels of SBP(*p* = 0.002), DBP(*p* < 0.001), and TG(*p* < 0.001) and higher levels of HDL(*p* < 0.001) than men.

**Table 1:** Characteristics of the participants at baseline and at follow-up by age groups: Population-based retrospective study of Japan from 2008 to 2017 in Japan

**variables**		**Men *n* = 230 (30.8%)**					
		***n***	**2008 year**	**95% CI**	**2017 year**	**95% CI**	***p***
age (years)			56.8 ± 7.0		66.8 ± 7.0		
Height (cm)	all	230	167.4 ± 6.1	166.6–168.1	166.4 ± 6.3	165.6–167.2	.078
	40 years	43	169.3 ± 5.0	167.7–170.8	168.5 ± 5.0	166.9–170.0	.460
	50 years	86	168.4 ± 6.3	167.0–169.7	167.4 ± 6.3	166.0–168.7	.309
	60 years	101	165.7 ± 6.1	164.5–166.9	164.7 ± 6.4	163.3–165.8	.190
BW (kg)	all	230	65.8 ± 10.2	64.5–67.2	64.9 ± 10.3	63.6–66.2	.003
	40 years	43	69.4 ± 13.5	65.2–73.5	68.8 ± 13.0	64.8–72.4	.544
	50 years	86	67.4 ± 8.2	65.6–69.1	66.2 ± 9.0	64.3–68.2	.010
	60 years	101	63.0 ± 9.4	61.1–64.9	62.1 ± 9.3	60.3–64.0	.042
WC (cm)	all	230	84.6 ± 8.5	83.5–85.7	84.4 ± 9.3	83.2–85.7	.657
	40 years	43	85.2 ± 10.8	81.9–88.5	86.0 ± 12.3	82.2–89.8	.509
	50 years	86	85.6 ± 7.0	84.2–87.2	84.7 ± 8.4	84.2–87.2	.096
	60 years	101	83.4 ± 8.5	81.7—85.0	83.5 ± 8.5	81.2–85.0	.816
SBP (mmHg)	all	230	124.1 ± 17.5	121.8–126.4	130.4 ± 18.6	128.0–132.8	.000
	40 years	43	120.8 ± 13.1	116.7–124.8	127.6 ± 16.1	122.7–132.6	.014
	50 years	86	127.7 ± 20.2	123.4–132.0	127.6 ± 16.2	127.2–135.8	.051
	60 years	101	122.5 ± 16.3	119.3–125.8	130.6 ± 18.5	127.0–134.3	< .001
DBP (mmHg)	all	230	76.4 ± 10.6	75.0–77.8	78.0 ± 11.2	76.6–79.4	.053
	40 years	43	73.8 ± 10.4	70.6–77.0	79.0 ± 11.1	75.6–82.5	.017
	50 years	86	78.9 ± 11.1	76.5–81.3	80.1 ± 12.5	77.4–82.8	.402
	60 years	101	75.4 ± 9.9	73.4–77.3	75.7 ± 9.7	73.8–77.6	.743
TG (mg/dl)	all	230	126.8 ± 70.4	117.7–136.0	124.1 ± 86.5	112.9–135.3	.590
	40 years	43	147.8 ± 100.9	116.7–179.0	144.9 ± 112.3	110.4–179.5	.825
	50 years	86	129.9 ± 60.7	116.9–143.0	121.2 ± 67.7	106.6–135.7	.165
	60 years	101	115.2 ± 60.0	103.4–127.1	117.7 ± 87.7	100.4–135.0	.771
HDL (mg/dl)	all	230	59.8 ± 16.4	57.6–61.9	61.3 ± 16.4	59.2–63.5	.038
	40 years	43	56.8 ± 13.4	52.7–60.9	60.6 ± 16.9	55.4–65.7	.046
	50 years	86	60.9 ± 17.3	57.2–64.6	61.8 ± 16.5	58.3–65.4	.476
	60 years	101	60.8 ± 16.8	56.7–63.4	61.2 ± 16.5	58.0–64.5	.262
LDL (mg/dl)	all	230	130.0 ± 30.2	126.1–133.9	129.2 ± 31.0	125.1–133.2	.647
	40 years	43	131.9 ± 29.7	122.8–141.1	130.2 ± 29.2	121.3–139.2	.700
	50 years	86	134.8 ± 30.1	128.4–141.3	131.4 ± 32.7	124.4–138.4	.223
	60 years	101	125.0 ± 29.9	119.1–130.9	126.9 ± 30.4	120.8–132.9	.473
Smoking status (Yes)		232	78 (33.9%)		55 (23.9%)		< .001
Drinking alcohol (Yes)		227	92 (40.4%)		88 (38.4%)		.595
Regular exercise (Yes)		229	79 (34.3%)		95 (41.5%)		.047
Skipping breakfast (Yes)		228	41 (17.9%)		28 (12.2%)		.043
**variables**		**Women *n* = 515(69.1%)**					
		***n***	**2008 year**	**95% CI**	**2017 year**	**95% CI**	***p***
age (years)			58.3 ± 6.3		68.3 ± 6.3		
Height (cm)	all	515	154.4 ± 5.5	153.9–154.9	153.3 ± 5.8	152.8–153.8	.002
	40 years	65	158.7 ± 4.9	156.5–158.9	157.4 ± 5.2	156.1–158.7	.618
	50 years	171	155.3 ± 5.8	154.4–156.2	154.2 ± 6.1	153.1–155.1	.098
	60 years	279	153.1 ± 5.0	152.5–153.7	151.8 ± 5.1	151.2–152.4	.003
BW(kg)	all	515	51.5 ± 8.5	50.9–52.1	50.7 ± 7.6	50.0–51.4	< .001
	40 years	65	53.7 ± 7.2	51.9–55.4	53.2 ± 8.3	51.2–55.3	.464
	50 years	171	52.2 ± 7.7	51.0–53.4	51.4 ± 7.9	50.2–52.6	.007
	60 years	279	50.6 ± 6.7	49.8–51.4	49.7 ± 7.2	48.9–50.6	< .001
WC (cm)	all	515	79.1 ± 8.5	78.4–79.8	79.9 ± 8.9	79.1–80.7	.007
	40 years	65	77.2 ± 8.2	75.2–79.2	78.9 ± 9.5	76.5–81.2	.066
	50 years	171	78.2 ± 9.0	76.8–79.6	79.3 ± 8.8	78.0–80.6	.040
	60 years	279	80.1 ± 8.2	79.1–81.1	80.5 ± 8.8	79.5–81.6	.287
SBP (mmHg)	all	515	119.8 ± 16.6	118.4–121.3	124.6 ± 18.6	123.0–126.3	< .001
	40 years	65	115.9 ± 12.4	112.8–119.0	117.1 ± 18.9	112.4–121.8	.532
	50 years	171	117.7 ± 15.6	115.4–120.1	123.1 ± 16.0	120.6–125.5	< .001
	60 years	279	122.0 ± 17.8	120.0–124.1	127.4 ± 19.5	125.1–129.7	< .001
DBP (mmHg)	all	515	71.8 ± 10.0	70.9–72.7	72.3 ± 10.6	71.3–73.2	.355
	40 years	65	70.8 ± 10.0	68.3–73.3	71.7 ± 13.5	68.3–75.0	.558
	50 years	171	71.6 ± 9.6	70.2–73.1	72.6 ± 10.4	71.0–75.0	.208
	60 years	279	72.2 ± 10.2	70.9–73.4	72.2 ± 10.0	71.0–73.4	.970
TG (mg/dl)	all	515	98.8 ± 48.5	94.6–103.0	96.1 ± 46.2	92.1–100.1	.175
	40 years	65	80.5 ± 39.4	70.7–90.2	86.1 ± 45.5	74.8–97.3	.375
	50 years	171	101.5 ± 49.5	94.0–109.0	97.3 ± 45.4	90.5–104.2	.206
	60 years	279	101.4 ± 49.1	95.6–107.1	97.9 ± 46.7	92.2–103.2	.167
HDL (mg/dl)	all	515	73.1 ± 18.3	71.5–74.7	73.1 ± 17.1	71.7–74.6	.038
	40 years	65	71.6 ± 18.3	67.0–76.1	75.3 ± 18.9	70.6–79.9	.038
	50 years	171	73.8 ± 19.6	70.8–76.7	62.9 ± 18.0	70.2–75.6	.381
	60 years	279	73.1 ± 17.4	71.1–75.2	72.8 ± 16.2	70.9–74.7	.624
LDL (mg/dl)	all	515	134.3 ± 29.2	131.8–136.8	135.9 ± 28.5	133.4–138.4	.647
	40 years	65	112.7 ± 26.4	106.1–119.2	128.8 ± 31.7	121.0–136.7	< .001
	50 years	171	136.3 ± 27.8	132.1–140.5	138.6 ± 27.3	134.5–142.7	.236
	60 years	279	138.1 ± 28.6	134.8–141.5	135.9 ± 28.2	132.6–139.2	.138
Smoking status (Yes)		515	45 (8.7%)		29 (5.7%)		.001
Drinking alcohol (Yes)		507	61 (11.9%)		50 (9.8%)		.165
Regular exercise (Yes)		507	175 (34.1%)		221 (43.4%)		< .001
Skipping breakfast (Yes)		506	56 (10.9%)		40 (7.9%)		.093

Characteristics are expressed as means ± standard deviations for continuous variables and n (%) for categorical variables. *P*-values were derived from paired t-tests for continuous variables and McNemar tests for categorical variables

*BW* body weight, *WC* waist circumference, *DBP* diastolic blood pressure, *SBP* systolic blood pressure, *TG* triglycerides, *HDL* high-density lipoprotein cholesterol, *LDL-C* low-density lipoprotein cholesterol

Among the study participants, 33.9% of the men and 7.8% of the women smoked, also 40.4% of the men and 11.9% of the women drunk alcohol every day. In contrast, there was no sex difference in exercise patterns.

Regarding characteristics at age at baseline, the mean body weight of participants in their 60 s had 63.0 ± 9.4 for men and 50.6 ± 6.7 for women, a lower for both sexes as compared to the other age groups. Women in their 60 s had a wider distribution of WC as well as higher SBP and DBP than did younger women, although this trend was not observed in men. Men in their 60 s had lower TG and LDL levels than men in their 40 s, but women in their 60 s had higher TG and LDL levels than did women in their 40 s.

The changes in anthropometric and metabolic biomarkers during the 10-year follow-up period are shown in Table 1. Body weight decreased 0.8 kg (*p* < 0.001) for women, 0.9 kg (*p* = 0.003) for men, decreased in all age groups in both sexes. There was a significant difference both men and women except in their 40 s. Waist circumference increased 0.8 cm (*p* = 0.007) for women, 0.2 cm (*p* = 0.657) for men. WC increased, except among men in their 50 s. There was no significant difference in any age group. SBP increased in all age groups for both sexes, with the largest increase in participants in their 60s. DBP increased in all age groups; however, this was non-significant.

Changes in TG, HDL, and LDL levels varied by age group. In women, TG and HDL levels increased in their 40 s but decreased in older age groups. However, this trend was not observed in men.

**Fig. 2:**
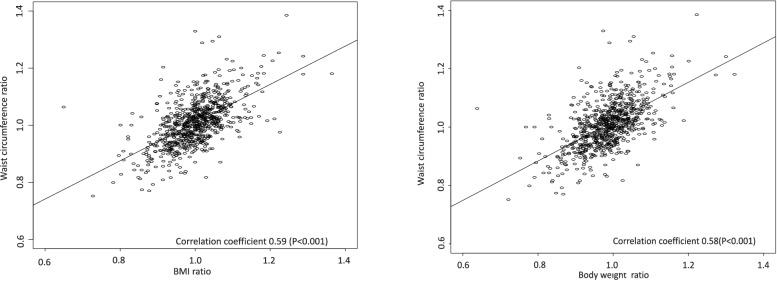
Correlation of Waist circumference ratio with BMI ratio and Body weight ratio: Population-based retrospective study of Japan from 2008 to 2017 in Japan

Figure 2 shows the correlation of the 10-year change ratio of WC with the change ratio of BMI as well as the change ratio of body weight. Changes in WC were moderately correlated with changes in BMI (*r* = 0.59, *p* < 0.001) and body weight (*r* = 0.58, *p* < 0 0.001).

GLM was used to estimate the changes in WC (Table 2). A significant positive association was found between WC and sex. However, there was no significant association between WC and any of the lifestyle factors such as smoking status, drinking alcohol, skipping breakfast, or regular exercise. Changes in TG, HDL, and LDL levels during the follow-up period are shown in Table 2. A significant positive association with sex and waist ratio was found with changes in TG and LDL levels.

**Table 2:** Regression coefficient between the change in WC and factors related to WC increase by generalized linear models: Population-based retrospective study of Japan from 2008 to 2017 in Japan

Dependent variables	Independent variables	B	SE	95% CI	*p*
WC	Intercept	4.386	0.013	4.36–4.41	< .001
	Sex	0.062	0.010	0.04–0.08	< .001
	Age	0.010	0.026	-0.04–0.06	.710
	Baseline Waist	-0.015	0.010	-0.03–0.00	.134
	Smoking status	-0.010	0.014	-0.04–0.02	.475
	Regular exercise	-0.006	0.009	-0.02–0.01	.500
	Skipping breakfast	-0.007	0.019	-0.04–0.03	.720
	Drinking alcohol	-0.017	0.012	-0.04–0.01	.147
AIC 5410.2					
TG	Intercept	4.068	0.281	3.53–4.61	< .001
	Sex	0.272	0.043	0.19–0.36	< .001
	Age	-0.117	0.128	-0.36–0.13	.359
	Baseline Waist	-0.012	0.052	-0.12–0.09	.815
	Waist ratio	0.570	0.263	0.06–1.07	.030
AIC 8307.7					
HDL	Intercept	4.473	0.113	4.25–4.70	.001
	Sex	-0.177	0.021	-0.22–-0.14	< .001
	Age	-0.053	0.055	-0.16–0.06	.335
	Baseline Waist	0.009	0.021	-0.03–0.05	.683
	Waist ratio	-0.147	0.107	-0.36–0.06	.169
AIC 6351.3					
LDL	Intercept	4.694	0.103	4.50–4.89	< .001
	Sex	-0.045	0.018	-0.08–-0.01	.012
	Age	0.067	0.050	-0.03–0.16	.184
	Baseline Waist	-0.022	0.020	-0.06–0.02	.261
	Waist ratio	0.195	0.096	0.01–0.38	.043
AIC 7151.9					

*WC* waist circumference, *TG* triglycerides, *HDL* high-density lipoprotein cholesterol, *LDL-C* low-density lipoprotein cholesterol

**Fig. 3:**
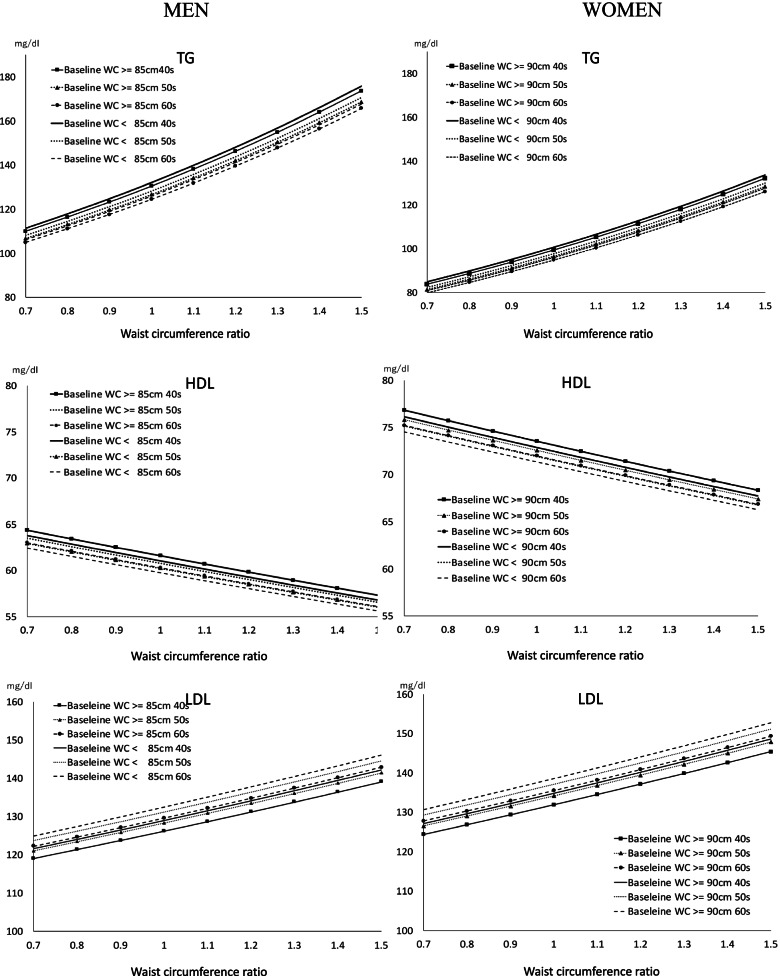
Scatter plots of WC changes and predicted values of TG, HDL, and LDL changes.: Population-based retrospective study of Japan from 2008 to 2017 in Japan. bWC, waist circumference; TG, triglycerides; HDL, high-density lipoprotein cholesterol; LDL, low-density lipoprotein cholesterol; Baseline WC, waist circumference at 2007; 40 s, 40 years; 50 s, 50 years; 60 s, 60 years

Figure 3 shows the predicted values of TG, HDL, and LDL from the test value prediction model formulas obtained by GLM analysis. The calculation results predicted that TG, HDL, and LDL would be 146.3 mg / dl, 59.8 mg / dl, and 131.1 mg / dl when WC increased 1.2 times in men in their 40 s whose baseline WC was less than 85 cm, an increase in WC could predict adverse changes in TG, HDL, and LDL levels.

For the same WC ratio of 1.2, the predicted values for TG showed 148.1 mg/dl for men and 112.8 mg/dl for women, which was higher predicted values for men, while the predicted values for HDL showed 58.0 mg/dl for men and 69.3 mg/dl for women, which lower predicted values for men. On the other hand, the predicted value of LDL showed 137.5 mg/dl in men and 144.0 mg/dl in females, which was higher in women.

## Discussion

This study focused on the changes in WC, lifestyle habits, and metabolic risks in the same population-based cohort for 10 years, and we investigated the relationship between 10-year changes in WC and lifestyle habits, including smoking status, alcohol consumption, regular exercise habits, and skipping breakfast. The effect of changes in waist circumference on metabolic risk in this population was estimated using model equations. Regardless of lifestyle habits, the average body weight for both men and women was significantly reduced over the 10 years, whereas WC increased. An increase in WC was associated with estimated levels of TG, HDL, and LDL, which were worse after 10 years.

Cross-sectional and longitudinal studies have shown that WC and weight increase with age [23–26]. In particular, women have greater increases than men of the same age regardless of race [5, 27]. A 20-year follow-up study of Japanese women showed no change in weight and an increase in WC after 20 years [28]. In a previous study of Japanese, weight loss in Japanese was associated with aging, consistent with the trend of weight loss and increase in waist circumference with aging in this study.

Many studies have shown an association between lifestyle and obesity [29–32]. In this study, lifestyle did not affect the change in waist circumference. It has been found that smoking is associated with visceral fat accumulation, abnormal glucose metabolism, and more than lipid metabolism [33]. On the other hand, smoking cessation has also been found to increase body fat [28, 29]. Smoking cessation promotes food and calorie intake and reduce basal metabolism owing to the reduced energy needed for nicotine metabolism, and the resultant body weight gain is attributable to increased body fat [34]. Smokers who ceased smoking had a significant increase in body weight [29]. More than Five years or more after quitting smoking, the prevalence of metabolic syndrome was similar to that of nonsmokers [35]. In this study, smoking did not show any effect on waist circumference. For women, the effect was probably due to the small number of smokers, and for men, the small number who quit smoking over a 10-year period.

With regard to alcohol, different results have been shown for alcohol intake and weight gain [36]. In addition, it appears that the association between skipping breakfast and obesity differs between children and adults. However, the recent review study showed an association between breakfast skipping and obesity [37], and a cross-sectional study on breakfast skipping and abdominal obesity in children found an association. Obesity is determined by the imbalance of energy intake and energy expenditure [38]. This study did not investigate the amount alcohol consumption or breakfast intake. In the population of this study, daily alcohol intake was about 40% in males and 10% in women, and breakfast skippers were around 10% in both males and women. In this population, it is unlikely that skipping breakfast and daily alcohol intake are factors in increasing waist circumference. In the future, it will be necessary to consider the amount of alcohol consumed and the amount and content of breakfast.

It is known that waist circumference decreases with exercise [39]. In this study, no change in waist circumference due to the exercise was observed. The current questionnaire asked about regular exercise habits. The exercise habit in this study was about 40% for both men and women, and few of them exercised. Even without exercise habits, the amount of activity in daily life, such as walking and stretching more often than usual, may have affected the results. On the other hand, weight decreased with age in this study. Weight loss is influenced by muscle loss, which begins to decrease around age 40 [40]. It is said that resistance training is effective in preventing muscle loss in elderly and obese women [41]. In particular, the higher the weekly training frequency, the more effective it is said to be. In the present study, many people did not practice regular exercise.

In this study, the effect of changes on abdominal circumference was not evident in every single lifestyle. It is thought that aging and years of complex lifestyle habits on abdominal circumference may have an effect. However, since abdominal circumference increases with age and the effect on lipid metabolism is presumed, health management focusing on abdominal circumference is important for the elderly.

This study had several limitations. First, this was a retrospective cohort study, and caution is required when interpreting the results. In the future, it will be necessary to conduct prospective cohort studies to provide more reliable results. Second, as our sample comprised middle-aged and older adults from a relatively small community in Japan, the generalizability of the results is limited, and the results cannot be applied to age groups including working people and other races. Lifestyle-related differences may exist between these groups. Third, the inclusion of participants who underwent health checkups may have produced a selection bias toward highly health-conscious people. Fourth, for lifestyle-related variables, the questionnaire used was from the Specialized Health Examination conducted by the Japanese government; therefore, lifestyle habits were analyzed using binary variables. The analyses of the categorical data on the lifestyle variables did not provide sufficient power to achieve statistical significance compared with those of continuous data. In the future, it will be necessary to conduct an analysis using indicators that can evaluate the content of lifestyle habits in more detail. Despite these limitations, the use of the 10-year follow-up data in the current study may be useful in identifying changes in WC trajectories over time.

## Conclusions

The results of this study showed that WC increased in both men and women aged 40–65 years during the 10-year period despite the decrease in body weight. Although there was no statistical relationship between the increase in WC and lifestyle habits, the results showed that laboratory values related to lipid metabolism increased as WC increased. The results suggest that WC gain with age in place of body weight should be focused during health checkups to prevent lifestyle-related diseases.

## Data Availability

The data that support the findings of this study are available from the corresponding author, upon reasonable request.

## Abbreviations

WC: Waist circumference
BMI: Body mass index
HDL: High-density lipoprotein
TG: Triglycerides
SBP: Systolic blood pressure
DBP: Diastolic blood pressure
LDL: Low-density lipoprotein
GLM: Generalized linear model

## References

[1] Kohro T, Furui Y, Mitsutake N (2008). The Japanese national health screening and intervention program aimed at preventing worsening of the metabolic syndrome. Int Heart J.

[2] Ministry of Health, Labour and Welfare. The National Health and Nutrition Survey in Japan. Tokyo, Japan. WWW document. 2019. URL http://www.mhlw.go.jp/stf/seisakunitsuite/bunya/kenkou_iryou/kenkou/eiyou/r1-houkoku_00002.html. Accessed 29 Sep 2021.

[3] Garn SM, Leonard WR, Hawthorne VM (1986). Three limitations of the body mass index. Am J Clin Nutr.

[4] Gallagher D, Visser M, Sepulveda D, Pierson RN, Harris T, Heymsfield SB (1996). How useful is body mass index for comparison of body fatness across age, sex, and ethnic groups?. Am J Epidemiol.

[5] Cheong KC, Ghazali SM, Hock LK (2015). The discriminative ability of waist circumference, body mass index and waist-to-hip ratio in identifying metabolic syndrome: Variations by age, sex and race. Diabetes Metab Syndr.

[6] Rothman KJ (2008). BMI-related errors in the measurement of obesity. Int J Obes (Lond).

[7] Kuk JL, Saunders TJ, Davidson LE, Ross R (2009). Age-related changes in total and regional fat distribution. Ageing Res Rev.

[8] Zamboni M, Armellini F, Sheiban I, Marchi De (1992). Relation of body fat distribution in men and degree of coronary narrowings in coronary artery disease. Am J Cardiol.

[9] Folsom AR, Kaye SA, Sellers TA (1993). Body fat distribution and 5-year risk of death in older women. JAMA.

[10] Klein S, Allison DB, Heymsfield SB (2007). Waist Circumference and Cardiometabolic Risk: a Consensus Statement from Shaping America’s Health: Association for Weight Management and Obesity Prevention; NAASO, the Obesity Society; the American Society for Nutrition; and the American Diabetes Association. Obesity (Silver Spring).

[11] Ross R, Neeland IJ, Yamashita S (2020). Waist circumference as a vital sign in clinical practice: a Consensus Statement from the IAS and ICCR Working Group on Visceral Obesity. Nat Rev Endocrinol.

[12] de Hollander EL, Bemelmans WJ, Boshuizen HC (2012). The association between waist circumference and risk of mortality considering body mass index in 65- to 74-year-olds: a meta-analysis of 29 cohorts involving more than 58 000 elderly persons. Int J Epidemiol.

[13] Albrecht SS, Gordon-Larsen P, Stern D, Popkin BM (2015). Is waist circumference per body mass index rising differentially across the United States, England, China and Mexico?. Eur J Clin Nutr.

[14] Elobeid MA, Desmond RA, Thomas O, Keith SW, Allison DB (2007). Waist circumference values are increasing beyond those expected from BMI increases. Obesity (Silver Spring).

[15] Freedman DS, Ford ES (2015). Are the recent secular increases in the waist circumference of adults independent of changes in BMI?. Am J Clin Nutr.

[16] Visscher TL, Heitmann BL, Rissanen A, Lahti-Koski M, Lissner L (2015). A break in the obesity epidemic? Explained by biases or misinterpretation of the data?. Int J Obes (Lond).

[17] Walls HL, Stevenson CE, Mannan HR (2011). Comparing trends in BMI and waist circumference. Obesity (Silver Spring).

[18] Sugihara M, Oka R, Sakurai M (2011). Age-related changes in abdominal fat distribution in Japanese adults in the general population. Intern Med.

[19] Johnson W, Norris T, Hamer M (2021). Secular changes in mid-adulthood body mass index, waist circumference, and low HDL cholesterol between 1990, 2003, and 2018 in Great Britain. Eur J Clin Nutr.

[20] Gallardo-Alfaro L, Bibiloni MDM, Mascaro CM, Montemayor S, Ruiz-Canela M, Salas-Salvado J, et al. Leisure-Time Physical Activity, Sedentary Behaviour and Diet Quality are Associated with Metabolic Syndrome Severity: The PREDIMED-Plus Study. Nutrients. 2020;12(4). 10.3390/nu12041013. 10.3390/nu12041013 PMC7230557 32272653

[21] Ministry of Health, Labour and Welfare. Standard Health Screening and Health Guidance Program. Tokyo, Japan. WWW document. 2018. URL http://www.mhlw.go.jp/stf/seisakunitsuite/bunya/0000194155.html. Accessed 02 May 2021.

[22] R Core Team. R: A language and environment for statistical computing. R Foundation for Statistical Computing. Vienna, Austria. WWW document. 2018. URL http://www.R-project.org/. Accessed 02 Jul 2018.

[23] Carmelli D, McElroy MR, Rosenman RH (1991). Longitudinal changes in fat distribution in the Western Collaborative Group Study: a 23-year follow-up. Int J Obes.

[24] Zamboni M, Zoico E, Scartezzini T (2003). Body composition changes in stable-weight elderly subjects: the effect of sex. Aging Clin Exp Res.

[25] Hughes VA, Roubenoff R, Wood M, Frontera WR, Evans WJ, Fiatarone Singh MA (2004). Anthropometric assessment of 10-y changes in body composition in the elderly. Am J Clin Nutr.

[26] Arabshahi S, Lahmann PH, Williams GM, van der Pols JC (2014). Predictors of change in weight and waist circumference: 15-year longitudinal study in Australian adults. Eur J Clin Nutr.

[27] Ye M, Robson PJ, Eurich DT, Vena JE, Xu JY, Johnson JA (2019). Anthropometric changes and risk of diabetes: are there sex differences? A longitudinal study of Alberta’s Tomorrow Project. BMJ Open.

[28] Kume S, Tokumistu N, Sakamoto S, Hagiwara H (2012). Differences in lifestyle among Japanese women whose body shapes changed in different ways over 20 years. Transact Japanese Soc Med Biol Eng.

[29] Harris KK, Zopey M, Friedman TC (2016). Metabolic effects of smoking cessation. Nat Rev Endocrinol.

[30] Sumi M, Hisamatsu T, Fujiyoshi A (2019). Association of Alcohol Consumption With Fat Deposition in a Community-Based Sample of Japanese Men: The Shiga Epidemiological Study of Subclinical Atherosclerosis (SESSA). J Epidemiol.

[31] Sakurai M, Yoshita K, Nakamura K (2017). Skipping breakfast and 5-year changes in body mass index and waist circumference in Japanese men and women. Obes Sci Pract.

[32] Lee IM, Djousse L, Sesso HD, Wang L, Buring JE (2010). Physical activity and weight gain prevention. JAMA.

[33] Komiya H, Mori Y, Yokose T (2006). Smoking as a risk factor for visceral fat accumulation in Japanese men. Tohoku J Exp Med.

[34] Moffatt RJ, Owens SG (1991). Cessation from cigarette smoking: changes in body weight, body composition, resting metabolism, and energy consumption. Metabolism.

[35] Ishizaka N, Ishizaka Y, Toda E (2007). Relationship between smoking, white blood cell count and metabolic syndrome in Japanese women. Diabetes Res Clin Pract.

[36] Traversy G, Chaput JP (2015). Alcohol Consumption and Obesity: An Update. Curr Obes Rep.

[37] Ma X, Chen Q, Pu Y (2020). Skipping breakfast is associated with overweight and obesity: A systematic review and meta-analysis. Obes Res Clin Pract.

[38] Endalifer ML, Diress G (2020). Epidemiology, Predisposing Factors, Biomarkers, and Prevention Mechanism of Obesity: A Systematic Review. J Obes.

[39] Cardenas Fuentes G, Bawaked RA, Martinez Gonzalez MA (2018). Association of physical activity with body mass index, waist circumference and incidence of obesity in older adults. Eur J Public Health.

[40] Ochi M, Kohara K, Tabara Y (2010). Arterial stiffness is associated with low thigh muscle mass in middle-aged to elderly men. Atherosclerosis.

[41] Toselli S, Badicu G, Bragonzoni L, Spiga F, Mazzuca P, Campa F. Comparison of the Effect of Different Resistance Training Frequencies on Phase Angle and Handgrip Strength in Obese Women: a Randomized Controlled Trial. Int J Environ Res Public Health. 2020;17(4). 10.3390/ijerph17041163. 10.3390/ijerph17041163 PMC7068258 32059579

